# Supplementary material for: The role of livelihood diversification in agricultural modernization among tribal farmers in Bangladesh: A binary logistic model approach
Source: PLoS One. 2025 Feb 25;20(2):e0318457. doi: 10.1371/journal.pone.0318457 (PMC11856501; doi:10.1371/journal.pone.0318457)
Supplement: Attachment — Submitted filename: PONE-D-24-36026.pdf [file pone.0318457.s002.pdf]

PLOS ONE

Influence of livelihood diversification on the adoption of modern agricultural practices by tribal farmers in hilly areas of Bangladesh

--Manuscript Draft--

**Manuscript Number:**	PONE-D-24-36026
**Article Type:**	Research Article
**Full Title:**	Influence of livelihood diversification on the adoption of modern agricultural practices by tribal farmers in hilly areas of Bangladesh
**Short Title:**	Influence of livelihood diversification on the adoption of modern agricultural practices
**Corresponding Author:**	Md. Monirul Islam, PhD<br>Bangladesh Agricultural University<br>Mymensingh, BANGLADESH
**Keywords:**	Livelihood diversification; Adoption decision; Modern agricultural practices; Tribal farmers; Profitability analysis; Binary logistic regression model
**Abstract:**	This study aimed to evaluate the influence of livelihood diversification on tribal farmers' decision to embrace modern agricultural practices in Bangladesh. In addition to this, profitability from farm and non-farm sectors were also considered. Data were collected through a structured questionnaire during the month of February 2021 from 115 tribal farmers, followed by a simple random sampling technique. In the study area, only 9.57% of farmers were involved in crop cultivation. The study calculated that human labor cost was the highest in rice production (about 37%). The benefit-cost ratio (BCR) of rice production, livestock, and homestead gardening was estimated at 1.46, 1.41, and 1.09, respectively. Among all agricultural components practiced by the tribal farmers, rice production was more efficient than others. The regression findings showed that livelihood diversification, age of the farmers, access to information, household income, and farming experience had a significant correlation with the adoption decision of modern agricultural practices. Therefore, promoting livelihood diversification among tribal farmers is crucial for enhancing their resilience and income sustainability. This could include providing training, access to markets, and financial support for activities such as poultry and livestock rearing and non-farm enterprises like homestead gardening.
**Order of Authors:**	Anika Tasnim
	Jasim Uddin Ahmed, PhD
	Md. Jahangir Alam Khan, PhD
	Md. Abdullah Al-Noman
	Arifa Jannat, PhD
	Md. Monirul Islam, PhD
**Additional Information:**	
**Question**	**Response**
**Financial Disclosure**<br><br>Enter a financial disclosure statement that describes the sources of funding for the work included in this submission. Review the submission guidelines for detailed requirements. View published research articles from *PLOS ONE* for specific examples.	The author(s) received no specific funding for this work.

Powered by Editorial Manager® and ProduXion Manager® from Aries Systems Corporation

This statement is required for submission and **will appear in the published article** if the submission is accepted. Please make sure it is accurate.<br><br>**Funded studies**<br>Enter a statement with the following details:<br>• Initials of the authors who received each award<br>• Grant numbers awarded to each author<br>• The full name of each funder<br>• URL of each funder website<br>• Did the sponsors or funders play any role in the study design, data collection and analysis, decision to publish, or preparation of the manuscript?<br><br>Did you receive funding for this work?	
**Competing Interests**<br><br>Use the instructions below to enter a competing interest statement for this submission. On behalf of all authors, disclose any competing interests that could be perceived to bias this work—acknowledging all financial support and any other relevant financial or non-financial competing interests.<br><br>This statement is required for submission and **will appear in the published article** if the submission is accepted. Please make sure it is accurate and that any funding sources listed in your Funding Information later in the submission form are also declared in your Financial Disclosure statement.<br><br>View published research articles from *PLOS ONE* for specific examples.	The authors have declared that no competing interests exist.

**NO authors have competing interests**<br><br>Enter: *The authors have declared that no competing interests exist.*<br><br>**Authors with competing interests**<br><br>Enter competing interest details beginning with this statement:<br><br>*I have read the journal's policy and the authors of this manuscript have the following competing interests: [insert competing interests here]*<br><br>* typeset	
**Ethics Statement**<br><br>Enter an ethics statement for this submission. This statement is required if the study involved:<br><br>• Human participants<br>• Human specimens or tissue<br>• Vertebrate animals or cephalopods<br>• Vertebrate embryos or tissues<br>• Field research<br><br>Write "N/A" if the submission does not require an ethics statement.<br><br>General guidance is provided below. Consult the submission guidelines for detailed instructions. **Make sure that all information entered here is included in the Methods section of the manuscript.**	The study was approved by the Sylhet Agricultural University Research System (SAURES) in accordance with the rules and regulations of the Sylhet Agricultural University, Sylhet, Bangladesh.

Powered by Editorial Manager® and ProduXion Manager® from Aries Systems Corporation

**Format for specific study types**<br><br>**Human Subject Research (involving human participants and/or tissue)**<br>• Give the name of the institutional review board or ethics committee that approved the study<br>• Include the approval number and/or a statement indicating approval of this research<br>• Indicate the form of consent obtained (written/oral) or the reason that consent was not obtained (e.g. the data were analyzed anonymously)<br><br>**Animal Research (involving vertebrate animals, embryos or tissues)**<br>• Provide the name of the Institutional Animal Care and Use Committee (IACUC) or other relevant ethics board that reviewed the study protocol, and indicate whether they approved this research or granted a formal waiver of ethical approval<br>• Include an approval number if one was obtained<br>• If the study involved *non-human primates*, add *additional details* about animal welfare and steps taken to ameliorate suffering<br>• If anesthesia, euthanasia, or any kind of animal sacrifice is part of the study, include briefly which substances and/or methods were applied<br><br>**Field Research**<br><br>Include the following details if this study involves the collection of plant, animal, or other materials from a natural setting:<br>• Field permit number<br>• Name of the institution or relevant body that granted permission	
**Data Availability**<br><br>Authors are required to make all data underlying the findings described fully available, without restriction, and from the time of publication. PLOS allows rare exceptions to address legal and ethical concerns. See the PLOS Data Policy and FAQ for detailed information.	Yes - all data are fully available without restriction

A Data Availability Statement describing where the data can be found is required at submission. Your answers to this question constitute the Data Availability Statement and **will be published in the article**, if accepted.<br><br>**Important:** Stating 'data available on request from the author' is not sufficient. If your data are only available upon request, select 'No' for the first question and explain your exceptional situation in the text box.<br><br>Do the authors confirm that all data underlying the findings described in their manuscript are fully available without restriction?	
**Describe where the data may be found in full sentences. If you are copying our sample text, replace any instances of XXX with the appropriate details.**<br><br>• If the data are **held or will be held in a public repository**, include URLs, accession numbers or DOIs. If this information will only be available after acceptance, indicate this by ticking the box below. For example: *All XXX files are available from the XXX database (accession number(s) XXX, XXX.).*<br>• If the data are all contained **within the manuscript and/or Supporting Information files**, enter the following: *All relevant data are within the manuscript and its Supporting Information files.*<br>• If neither of these applies but you are able to provide **details of access elsewhere**, with or without limitations, please do so. For example:<br><br>*Data cannot be shared publicly because of [XXX]. Data are available from the XXX Institutional Data Access / Ethics Committee (contact via XXX) for researchers who meet the criteria for access to confidential data.*<br><br>*The data underlying the results presented in the study are available from (include the name of the third party*	All relevant data are within the manuscript and its Supporting Information files.

and contact information or URL).<br>• This text is appropriate if the data are owned by a third party and authors do not have permission to share the data.<br><br>* typeset	
Additional data availability information:	Tick here if the URLs/accession numbers/DOIs will be available only after acceptance of the manuscript for publication so that we can ensure their inclusion before publication.

Manuscript
Click here to access/download;Manuscript;Anika_paper-20240821.docx

Influence of livelihood diversification on the adoption of modern agricultural practices by tribal farmers in hilly areas of Bangladesh

Anika Tasnim[1], Jasim Uddin Ahmed[1], Md. Jahangir Alam Khan[2], Md. Abdullah Al-Noman[3], Arifa Jannat[4], Md. Monirul Islam[3*]

[1]Department of Agricultural Economics and Policy, Sylhet Agricultural University, Sylhet-3100, Bangladesh
[2]Director, Dhaka School of Economics, University of Dhaka
[3]Department of Agricultural Economics, Bangladesh Agricultural University, Mymensingh-2202, Bangladesh
[4]Institute of Agribusiness and Development Studies, Bangladesh Agricultural University, Mymensingh-2202, Bangladesh

*Correspondence: monir96@bau.edu.bd
ORCID ID: https://orcid.org/0000-0003-0045-3897

Abstract

This study aimed to evaluate the influence of livelihood diversification on tribal farmers' decision to embrace modern agricultural practices in Bangladesh. In addition to this, profitability from farm and non-farm sectors were also considered. Data were collected through a structured questionnaire during the month of February 2021 from 115 tribal farmers, followed by a simple random sampling technique. In the study area, only 9.57% of farmers were involved in crop cultivation. The study calculated that human labor cost was the highest in rice production (about 37%). The benefit-cost ratio (BCR) of rice production, livestock, and homestead gardening was estimated at 1.46, 1.41, and 1.09, respectively. Among all agricultural components practiced by the tribal farmers, rice production was more efficient than others. The regression findings showed that livelihood diversification, age of the farmers, access to information, household income, and farming experience had a significant correlation with the adoption decision of modern agricultural practices. Therefore, promoting livelihood diversification among tribal farmers is crucial for enhancing their resilience and income sustainability. This could include providing training, access to markets, and financial support for activities such as poultry and livestock rearing and non-farm enterprises like homestead gardening.

Keywords: Livelihood diversification; Adoption decision; Modern agricultural practices; Tribal farmers; Profitability analysis; Binary logistic regression model

1. Introduction

Indigenous people are uniquely identified because of their distinct culture, rites and rituals, dialect, and dresses from the larger community with whom they coexist. According to the United Nations International Children's Emergency Fund (UNICEF, 2019) 1,755,973 indigenous people are living in

Bangladesh whereas Chattogram has the highest concentration of indigenous people. The hilly areas of Chattogram are called Chattogram Hill Tracts (CHT), where education, communication, and medical facilities are rare, as well as poverty and illiteracy rates are high (Chakma & Maitrot, 2017). Indigenous people are mainly involved in agriculture, daily wage labor, and petty trade. Rozario et al. (2022) mentioned that these indigenous peoples in southeastern Bangladesh are highly vulnerable to natural calamities because of their poor livelihood standards. They have limited access to electricity, pure drinking water and sanitary facilities (Barau et al., 2019). Being one of the fastest-growing economies, Bangladesh has decreased its extreme poverty level from 82% in 1972 to 12.9% in 2016 (World Bank, 2017). However, this incredible achievement rarely touched this group of people due to a lack of accessibility and fewer opportunities to engage in alternative income-generating activities (Rasul & Gurung, 2024).

One of the most effective ways to fight poverty is to diversify one's sources of income, which involves moving from farming to non-farming industries as the rural non-farm economy grows (Start, 2001). Rural households undertake this process, termed rural livelihood diversification, to bolster their resilience and quality of life (Start, 2001; Ellis, 2000; Ellis, 1999). It is worth noting that indigenous peoples in the CHT are predominantly shifting cultivators (Nahar et al., 2020), and almost 70% rely on agriculture as a major or secondary source of income (Rasul, 2015), but their livelihood practices have varied slightly over time. There are two major types of cropland in CHT, those are plough and *jhum*[1] where 34% of households are involved in plough cropping only, 19% are involved in *jhum* only, and the rest of them do both plough and *jhum* agriculture (Barkat et al., 2017). A recent study shows that shifting cultivation is a form of land use among resource-poor communities with a rotation of cultivation and fallow in the same unit of land (Hossain, 2011). *Jhum* farming is changing fast in many regions, owing to population pressures as well as diversification of livelihood patterns to include permanent cash crop production and off-farm labor. However, due to the topographic characteristics, the people of CHT have limited access to livelihood diversification (Rasul & Gurung, 2024). Recent studies show that income and non-income poverty is widespread in the southeastern region of Bangladesh, and farmers of this area experience food shortages for two to six months a year (Barkat et al., 2017; Nath et al., 2005; Sen & Ali, 2015).

Nevertheless, modern agricultural practices have significantly changed the crop field by reducing dependency on human labor and saving the farmer's time (Gallardo & Sauer, 2018). The development of high-yielding varieties (HYVs) has flourished in a new era in the agricultural sector (Bokhtiar & Samsuzzaman, 2023). These improved practices are cost-effective as they notably increase the quality and quantity of yield (FAO, 2017). These practices can help the backward people to move toward a

[1] Jhum is a method of cultivation of different crops with rice on a hill slope. It is also known as the slash-and-burn method or Swedish cultivation.

more sustainable living. Acceptance of these technologies will provide farmers with a better subsistence.

Various studies have investigated the impacts of different livelihood activities on rural communities across diverse settings. For instance, Miah et al. (2012) underscored the substantial role of shifting cultivation and forest income in the livelihoods of the Chakma tribe in Bangladesh. Tailor et al. (2012) pinpointed challenges confronting tribal milk producers in Rajasthan, such as low productivity and limited access to green fodder. Xaba & Masuku (2013) analyzed factors affecting profitability in vegetable production, while Patr et al. (2014) assessed constraints in traditional pig production systems in Northeast India. T. Ahmed et al. (2015) divided into livelihood diversification patterns in rural Bangladesh, highlighting the significance of rice cultivation and entrepreneurship. Moreover, Martin & Lorenzen (2016) explored the motivations behind livelihood diversification in Laos, linking it to wealth status and asset ownership. Gautam & Andersen (2016) investigated how investing in high-return sectors influenced household well-being in Nepal, noting entry barriers leading to inequality. Mariyono (2018) examined factors shaping farmers' intentions to commercialize vegetable farming in Indonesia. Punitha et al. (2018) identified constraints to livelihood diversification as perceived by jumians in Northeast India. Jannat et al. (2019) scrutinized the impact of agricultural modernization on sustainable livelihoods in Bangladesh, emphasizing the importance of training in new technologies. All of these studies collectively highlight the crucial role of livelihood diversification in enhancing household welfare. While research on income diversification in Bangladesh has been extensive, there is a notable gap in studies focusing on tribal farmers in hilly areas, their agricultural practices, and socioeconomic conditions. Therefore, a study investigating livelihood diversification and its influence on adopting modern agricultural practices in hilly areas is warranted. To shed light on this issue, this study assesses the socioeconomic status of tribal households, evaluate earnings from diverse activities, analyze production practices, explore the impact of diversification on embracing modern agricultural methods, address their challenges, and propose coping strategies. The overall aim of the study was to find out the profitability of agricultural production practices, the extent of livelihood diversification, and the influence of livelihood diversification on the adoption of modern agricultural practices. The specific objectives were as follows: (a) to estimate the profitability of on-farm (crop and livestock production, fishing, or homestead gardening, etc.) and off-farm activities in the selected hilly areas; and (b) to document the influence of livelihood diversification on the adoption of modern agricultural practices decisions in the hilly areas.

2. Methodology

2.1 **Selection of the Study Area, Data Sources and Sampling Technique**

The study area was selected based on the objectives. This study was done on tribal farmers and CHT is where most tribal communities live. Rangamati is one of the districts of CHT, which has 10 upazilas; Baghaichari is one of them. Distance of Baghaichari is 118 Kilometers from *Rangamati Sadar*. Primary data were collected directly from farmers, and secondary data sources included authentic and dependable published articles, reports, journals, and documents. To estimate the sample size, the Yemane method was used, which scored 99.97 at a 10% precision level. A total of 115 samples were collected by using a simple random sampling technique. Data was collected from the farmers through direct interviews. Appropriate timing of data collection is equally important. The month February was chosen for data collection when *Boro* rice is grown in the farmer's field.

2.2 Analytical Techniques

2.2.1 Profitability analysis

The income from farm and non-farm activity was estimated by using profitability equations that determined net return, total variable costs, total fixed cost, Benefit-Cost Analysis (Undiscounted), etc. Profit is the difference between the value of goods and services produced by the farm and the costs of resources used in production.

$$NR = GR - (TVC + TFC) \dots \dots \dots (i)$$

where, NR = Total net return (Tk.); GR = Gross return (Tk.); TFC = Total fixed cost (Tk.); and TVC = Total variable cost (Tk.).

2.2.2 Simpson index of diversification (SID)

The income diversification sources were analyzed using the Simpson Index of Diversification (SID). The formula for the Simpson Index (Alam, 2004) is:

$$SID = 1 - \sum P_i^2 \, n_i \dots \dots .. (ii)$$

Where n is the total number of income sources and p_i is the income portion of i-th income source. The value of SDI falls between 0 and 1. The index's value is zero if there is just one source of income. As the number of sources increases, the shares (Pi) decline, as does the sum of the squared shares, so that SID approaches 1. Households with the most diversified income sources have the largest SID value and the least diversified income sources have the smallest SID value. The higher the number of income sources as well as the more evenly distributed the income shares, the higher the value of SID. The Simpson index of diversity was affected both by the number of income sources as well as by the distribution of income among different sources. Based on the SDI values, the level of

livelihood diversification was classified as (a) no diversification (SDI <= 0.01); (b) low level of diversification (SDI = 0.01-0.25); (c) medium level of diversification (SDI = 0.26-0.50); (d) high level of diversification (SDI = 0.51-0.75); and (e) very high level of diversification (SDI =>0.75).

In this study, respondents were asked to indicate the household's major livelihood activities such as crop production, livestock production, daily labor, small business, government and non-government jobs, etc. This question helped to understand the livelihood structure at the household level. Following past studies (Ellis, 1998), Livelihood activities were classified as on-farm livelihood activities comprising crop and livestock production, off-farm activities (which included earning wages for work on other farms), and non-farm activities where income was earned from non-agricultural sources such as job-holding and self-employment.

2.2.3 Factors influencing the adoption of modern agricultural practices

Following Wongnaa & Babu (2020), the binary logistic regression model was employed in analyzing the influence of livelihood diversification and other socio-economic variables on the adoption of modern agricultural practices decision of the farmers. Farmers may or may not respond fully or partially to modern agricultural practices or may not respond at all. This led to a binary dependent variable, making the logit model an appropriate model to be employed in this study.

The adoption model representing adoption behavior employed in this study follows the threshold theory of decision-making, proposed by Hill & Kau (1973). That is individuals with choices to make have reaction thresholds, which are determined by a multiplicity of factors. Choices of this nature are usually modeled as:

$$y_i = \beta_i x_i + u_i \qquad \text{(iii)}$$

where, x_1 = Livelihood Diversification Score (SDI); x_2 = Age in years; x_3 = Years of schooling; x_4 = Extension service; x_5= Access to information; x_6 = Credit; x_7 = Income; x_8 = Farming experience; y_i is equal to one if at least one modern agricultural practice is adopted by the farmer and zero otherwise.

$y_i = 1$, if, $x_i \geq$ x* and y_i = 0, if, xi <x* and x* is a critical value representing the joint effect of independent variables at the threshold level.

The decision to adopt a modern agricultural practice by a tribal farmer is binary, so a farmer may or may not adopt any of the practices. The model is binary, which involves estimating and examining the likelihood of adoption of modern agricultural practices, y, as a function of independent variables, x. For this study y_i therefore takes a value of 1 if a tribal farmer adopts at least one modern agricultural practice, and 0 if he/she does not adopt any of them. The estimation of such a qualitative response model allows us to estimate the conditional probability that y_i assumes one of the specified values.

Thus, the probability that a given tribal farmer will adopt at least one adaptation technology, $\beta_i x_i$> x* (y_i=1) is given as:

$$P_i = Prob\ (y_i = 1) = F\ (\beta' x_i) \qquad (iv)$$

The probability that a tribal farmer will not adopt any modern agricultural practices is, therefore, given as:

$$1 - P_i = Prob\ (y_i = 0) = 1 - F\ (\beta' x_i) \qquad (v)$$

where, y_i is the observed response of the *ith* observation of the response variable y and x_i is a set of explanatory variables of the *ith* respondent.

2.2.4 Problems faced by the tribal farmers

Problems faced by intermediaries in the study areas were measured by using structured questionnaires. The farmers were asked to give their opinions on selected problems that were identified during the data collection period. A four-point rating scale was used to compute the constraint score of a respondent. For each constraint score of 3, 2, 1 and 0 was assigned to indicate the extent of constraint as high, medium, low and not at all, respectively. The total constraint scores were computed for each respondent by adding his scores for all the constraints. The Constraint Facing Index was computed using the following formula (Afrad, 2002).

$$CFI = (C_h \times 3) + (C_m \times 2) + (C_l \times 1) + (C_n \times 0) \ldots\ldots..(vi)$$

Where, CFI = Constraints Facing Index; C_h= Number of respondents having high constraints; C_m= Number of respondents having medium constraints; C_l = Number of respondents having low constraints; and C_n= Number of respondents having no constraints.

2.2.5 Ethical approval

Ethical approval was sought and received from the Sylhet Agricultural University Research System (SAURES) in accordance with the rules and regulations of the Sylhet Agricultural University, Sylhet, Bangladesh. The type of approval was exempt because participation in the study did not expose the respondents to danger.

3. Results and Discussion

3.1 Socioeconomic Characteristics of the Tribal Farmers

Table 1 shows that most of the farmers (64.35%) belonged to the 15-49 age group, whereas 41 respondents (35.65%) belonged to the above 49 age category, and none was below 15. The findings demonstrate that most of the respondents were male (70.43%). The average family size is 4.91 in the study area, which is slightly higher than the country's average rural area household size of 4.11 (BBS, 2021). It was found that most of the families have 1-5 members (67.83%), and the rest, 32.17%,

belong to the 6-10 family members category. In the study area, most people took credit from the Bangladesh government social welfare program, [illegible]ee Bari Ektee Khamar', and a few took out loans from banks. Data shows that only 34.72% of tribal farmers can get extension services from the extension personnel. In the study area, 84.35% of respondents got access to the right information. Table 1 also shows that most respondents are marginal farmers (51.30%).

Table 1. Basic characteristics of the tribal farmers

Characteristics	No. of respondents	Percentage of respondents (%)
**Age categories**		
<15	0	0.0
15-49	74	64.35
>50	41	35.65
**Sex**		
Male	81	70.43
Female	34	29.56
**Family size**		
1-5	78	67.83
6-10	37	32.17
>10	0	0
1-5	78	67.83
**Level of education**		
Illiterate	46	40
Primary	35	30.43
Secondary	20	17.4
Above secondary	14	12.17
**Access to credit**		
No credit access	68	59.13
Credit access	47	40.87
**Access to extension service**		
Had access	40	34.78
No access	75	65.21
**Access to information**		
Had access	97	84.35
No access	18	15.65
**Farm Size**		
Landless (<.2ha)	18	15.65
Marginal (.2ha-.6ha)	59	51.30
Small (.6ha-1.008ha)	34	29.56
Medium (1.008ha-3.04ha)	4	3.48
Large (>3.04ha)	0	0
**Occupational patterns**		
Crop cultivation	11	9.57
Crop cultivation + Livestock rearing	11	9.57
Crop cultivation + non-farm	10	8.69
Crop cultivation + Poultry and livestock rearing	40	34.78
Crop cultivation + Poultry rearing + non-farm	8	6.95
Crop cultivation + Poultry and Livestock rearing + non-farm	19	16.52
Crop cultivation + Livestock rearing + non-	16	13.91

farm		
**Sources of Income Diversification**		
<0.01 (no diversification)	0	0
0.01-0.25 (low level)	17	14.78
0.26-0.50 (medium level)	28	24.35
0.51-0.75 (high level)	61	53.04
>0.75 (very high level)	9	7.82

Source: Author’s estimation based on field survey, 2021

In this study, all the respondents were crop cultivators. Besides that, their occupational pattern is significantly diversified. Table 1 demonstrates that 34.78% of farmers were engaged in Crop cultivation + Poultry and Livestock rearing. The number of sources of income is an important indicator of the livelihood diversification of a particular household. Researchers have used this technique (Saha & Bahal, 2010). [illegible]eholds having more sources of income are more diversified. Diversification is measured using the SID, which is categorized into five groups; up to 0.01 is considered no diversification. Between 0.01-0.25 is low, 0.26-0.50 is medium, 0.51-0.75 is high, and more than 0.75 is a very high level of diversification (Ahmed et al., 2015). The average Simpson index was 0.588. Table 1 also shows that most respondents (53.04%) were in a high-level diversified group. There was no respondent with zero Simpson Index. About 28 respondents (24.35%) had a medium level of diversification. Of the sampled households, 14.78% had a low, and 7.82% had a very high level of livelihood diversification.

3.2 Average Annual Income and Expenditure of the Tribal Farmers

Income is the most important indicator of people's socioeconomic status. The annual income of a family has been estimated based on all the year-round, and this study categorized the income of the household by the classification of Islam et al. (2018). The average total family income has been calculated by adding up the farm and non-farm sources of income during the study period. Figure 1 shows that only 4.34% are low-income people, 10.43% are medium-income people, and 85.22% are high-income people.

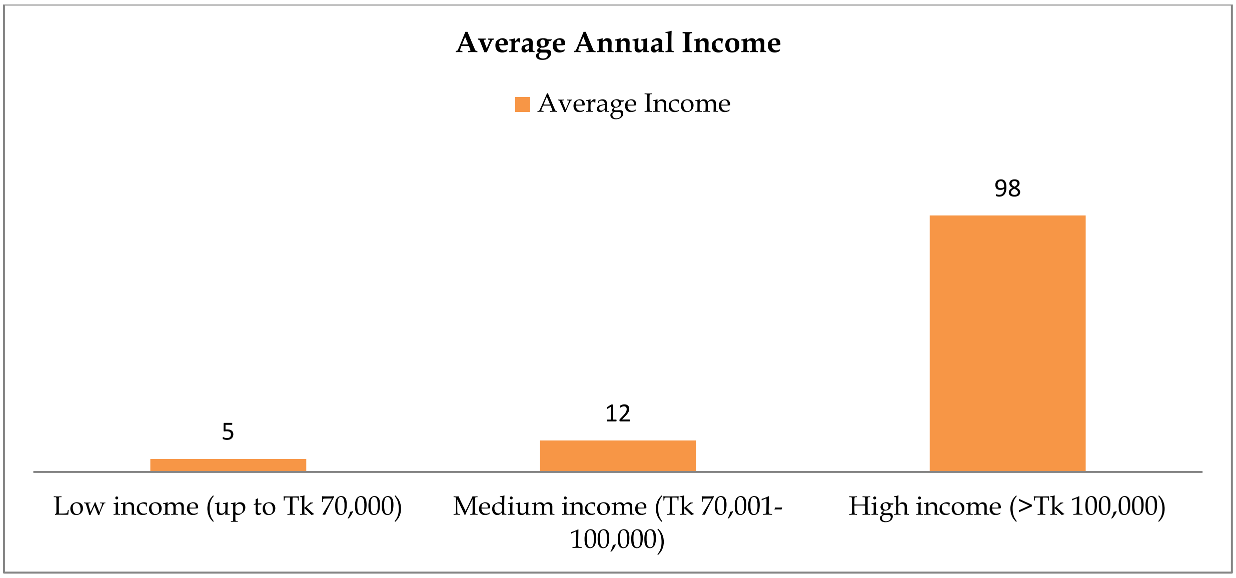

Figure 1[illegible]e annual income of the respondents

Average annual expenditure is another important factor to indicate the socioeconomic status of the respondents. The average annual expenditure of the respondents was classified into three groups, i.e., low, medium and high expenditure.

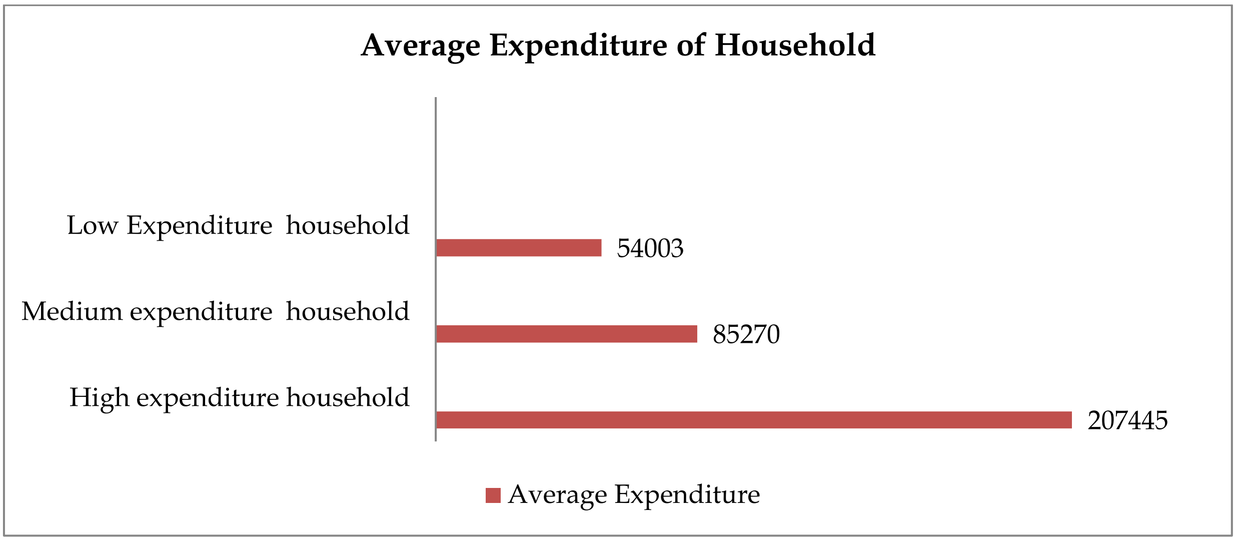

Figure 2. The annual average expenditure of the respondents

Figure 2 shows that around 6.08% of the respondents were in the low-expenditure category, 8.69% were in the medium-expenditure category, and 85.21% were in the high-expenditure category. Most of the respondents were in the high-expenditure category. Food items were the major expenditure items of the medium and high categories of expenditure groups. The total expenditure of the low, medium, and higher expenditure respondents was Tk. 54003, Tk. 85270, and Tk. 207445, respectively.

3.3 Adoption of Modern Agricultural Practices

Table 2 shows that tribal farmers also adopted modern technologies that have increased their production, reduced their costs, and made crop production profitable. HYVs are high-yielding, which attracted the farmers most (93.04%). Nearly 94.78% of farmers applied pesticides, and 89.57% of farmers applied fertilizers as well. However, some farmers (5.22%) were using an environment-friendly pest control method called 'perching.' The perching method hardly costs any[illegible]g. Chemical pesticides are comparatively expensive. Water pumps were playing crucial roles in irrigation. About 73.91% of farmers also mechanically harvest through reapers, which reduces their harvesting cost and risk of crop damage and boosts farmers' efficiency. No farmer was using seed fertilizer drills. Fruit orchard owners depended on the internet and used specialized apps to cure plant diseases. Commercial producers adopted developed fruit varieties.

Table 2. Adoption of modern agricultural practices

Items	Adopted	Not Adopted
HYVs	107 (93.04)	8 (6.96)
Use of Pesticides	109 (94.78)	6 (5.22)
Perching method	6 (5.22)	109 (94.78)
Use of chemical fertilizers	103 (89.57)	12 (10.43)
Artificial irrigation	86 (74.78)	29 (25.22)
Artificial dams	57 (49.57)	58 (50.43)
Use of tractors	103 (89.57)	12 (10.43)
Use of reaper	85 (73.91)	30 (26.09)
Seed fertilizer drills	0 (0.00)	115 (100.00)
Internet and apps	6 (5.22)	109 (94.78)

Source: Author's estimation based on field survey, 2021.

Note: Figure in the parenthesis indicates percentage.

3.4 Profitability Analysis

3.4.1 Cost and return from different agricultural components

Table 3 depicts the total cost of rice production per hectare is Tk. 76220.94, with the total variable cost at Tk. 62813.56, and the total fixed cost at Tk. 13407.38. The Gross return from one hectare of land was Tk. 111248.5. It is evident from Table 3 that the gross margin per hectare of the rice production is Tk. 48434, and the net margin per is Tk. 35027.56. Finally, the BCR (undiscounted) for rice production per hectare was 1.46, implying that 1.46 would be earned by every Tk. 1 investing in rice production. In livestock rearing, variable costs include feed costs, medicine facilities costs, artificial insemination costs, casual labor costs, etc. The total cost of rearing livestock per annum in the study area is Tk. 41614.16, where the total fixed cost is Tk. 6715.96, and the total variable cost is Tk. 34898.2. It can be seen from Table 4 that the annual gross return of livestock rearing in the study area is Tk. 58740. The analysis from Table 4 shows that the gross margin of rearing livestock per annum in the study area is Tk. 23842.07**.** Table 3 reveals that the annual net return of the sampled farmers for livestock rearing in the study area is Tk. 17126.11. Finally, the BCR for rearing livestock in the study area is 1.41. Homestead enterprise includes both vegetable farming and fruit orchards. Day by day, fruit orchards are getting more popular among tribal farmers.

Table 3. Profitability analysis of different agricult[illegible] components

**Cost and return items**	**Rice production (Tk./ha)**	**Livestock rearing (Tk./Year)**	**Homestead enterprise (Tk./Year)**
Total variable cost (A)	62813.56	34898.2	42308.09
Total fixed cost (B)	13407.38	6715.96	14476.34
Total cost (C)	76220.94	41614.16	56784.43
Gross return (D)	111248.5	58740.27	61933.45
Gross Margin (D-A)	48434.94	23842.07	19625.36
Net Return (E-B)	35027.56	17126.11	5149.02
BCR (Undiscounted)	1.46	1.41	1.09

Source: Author’s estimation based on field survey, 2021.

Jhum cultivation is an ancient method; however, it’s destructive to the environment. Several research has shown that agroforestry can be a better alternative to *jhum* cultivation. Table 3 represents the profitability of homestead enterprise which includes both vegetable farming and fruit orchards.

Human labor costs and irrigation costs are the highest among the variable costs. Variable cost is 74.51% whereas, fixed cost retains 25.89% of the total cost. BCR is 1.09 in this case.

3.5 Factors Influencing the Adoption of Modern Agricultural Practices

Livelihood diversification ($p < 0.05$) is significantly associated with adopting modern practices. The marginal effect shows that a one-unit increase in diversification value increases the probability of adopting modern agricultural practices by 114%. As diversification helps increase the probability of livelihood sustainability, adopting modern practices becomes easy for the farmers.

The age of the farmer had a significant correlation with the adoption decision ($p < 0.05$). The marginal effect of age indicates that a one-year increase in the farmer's age decreases the probability of adopting modern agricultural practices by 87.9%. This could be because young people are more concerned about adopting new technologies. As a result, a one-year increase in age decreases the probability of adoption in this context. Our findings are in line with previous studies, and it is evidenced that the age of the farmers significantly influences the adoption of agricultural technologies (Ahmed & Ahmed, 2023; Akudugu et al., 2023; Kabir & Rainis, 2015; Sarker et al., 2021; Wongnaa et al., 2018).

Access to information ($p < 0.05$) was found to have a positive and significant correlation with modern practices adoption in agriculture. The marginal effect shows that access to information increases the probability of adopting modern technology 2.134 times. The reason could be access to information highly influences farmers to switch to modern technologies. This is in line with the findings of Sarker et al., (2021), who found that access to information through extension personnel and farmers' field schools (FFS) would positively influence the adoption of new agricultural technologies. Along with this, Wu, (2022) found that accessing information online has a significant positive impact on family farms' adoption of new technologies.

Table 4. Factors influencing the adoption of modern agricultural practices

Variables	Coefficient	S.E.	Wald	p-value	ME
Constant	-2.634	0.749	11.839	0.001	0.076
Livelihood diversification (SID)	0.205**	0.037	6.903	0.034	1.14
Age	-0.031**	0.010	4.244	0.039	0.879
Years of schooling	0.028	0.048	0.635	0.425	1.029
Extension service (yes=1)	0.041	0.423	0.040	0.675	0.945
Access to information (yes=1)	0.777**	0.256	4.213	0.030	2.134
Access to credit (yes=1)	-0.067	0.345	0.056	0.767	0.734
Average annual income	0.452**	0.112	14.600	0.024	1.932

Farming experience	0.067***	0.020	14.344	0.000	1.343
Hosmer and Lemeshow Test	Chi-square= 4.260; p-value: 0.833				

Note: ME = Marginal Effect, * Significance level of 10%, ** Significance level of 5%, *** Significance level of 1%
Source: Author's estimation based on field survey, 2021.

The household income of a particular household is significantly associated with adoption decisions at a 5% significant level. The marginal effect of income indicates that a unit increase in income increases the probability of adopting modern agricultural practices by 193.2%. Increased household income increases the affordability of adopting modern agricultural practices. This study reveals that the average annual income of farmers is significantly associated with adoption decisions. Higher income levels generally provide farmers with the financial resources needed to invest in new technologies, purchase inputs, and manage risks associated with adopting innovative practices. Our findings are in line with previous studies, and it is evidenced that the farmers' average annual income inspires them to adopt new farm technologies (Wu, 2022). Farming experience ($p < 0.01$) is significantly associated with the adoption of modern agricultural practices. The marginal effect indicates that a one-year increase in farming experience increases the probability of adopting modern practices by 134.3%. There may be a reason behind this: with the increase in farming experience, farmers get a better understanding of the efficiency of different practices. The value of the chi-square test is 4.260, and the estimated p-value is 0.833, which is insignificant. It means the model is well-fitted. This finding is in line with a previous study, and it is evidenced that the farming experience of the farmers positively affects modern technology adoption (Sarker et al., 2021; Wongnaa et al., 2018). However, Ghosh et al. (2023) found no significant relationship between farming experience and the adoption of modern agricultural practices.

3.6. Problems Faced by the Tribal Farmers

In the study area, the computed value of CFI for the major problem was 238 out of a possible 345, with 23 respondents facing it to a high extent, 76 to a medium extent, and 16 to a low extent. The second-highest constraint was the product's price during harvesting, affected by an excessive supply that reduced prices to Tk. 19-20 per kg of paddy. High-interest rates were also significant, with a CFI of 228, ranked fourth, and affecting 21 farmers to a high extent, 73 to a medium extent, and 19 to a low extent. Small and fragmented land holdings, which discourage the adoption of new practices, were another issue with a CFI of 215 (Table 5).

Table 5. Constraints faced by the farmers.

Variables	CFI	Rank
Lack of sufficient credit supply	232	3
High-interest rate	228	4

Lack of knowledge about modern practices	97	10
Low price of product during harvesting	237	2
Fragmented land	215	5
Lack of human labor	0	13
Lack of suitable marketing facilities	214	6
Poor access to new technologies	238	1
Dominance of intermediaries	167	8
Disease attacks	135	9
Loss of product due to theft	24	12
The burden of old debt	71	11
Natural calamities	175	7

Source: Author’s estimation based on field survey, 2021

This constraint was faced by 35 respondents to a high extent, 32 to a medium extent, and 46 to a low extent. Geographical challenges in hill tract areas and tribal farmers' issues had a CFI of 214, affecting 33 farmers to a high extent and 54 to a medium extent. Natural calamities were a significant threat, with a CFI of 175, impacting 8 respondents to a high extent, 49 to a medium extent, and 53 to a low extent. The dominance of intermediaries, ranked eighth, had a CFI of 167, with 8 farmers affected to a high extent and 55 to a medium extent. Disease attacks, though not severe, had a CFI of 135, affecting 3 respondents to a high extent and 16 to a medium extent. Limited access to information due to illiteracy was an issue, with a CFI of 97, and affected 9 farmers to a high extent and 5 to a medium extent. Old debt was less significant, with a CFI of 71, impacting 3 farmers to a high extent and 15 to a medium extent. Product loss due to theft, mentioned by fruit orchard farmers, had a CFI of 24. Labor was not a constraint in the study area, with male labor costing 300TK/day and female labor 250TK/day, and a CFI of 0.

4. Conclusion and Policy Implications

The study explored livelihood diversification and its impact on the adoption of modern agricultural techniques. The results indicated that the decision to adopt modern agricultural practices was significantly correlated with livelihood diversification, the age of the farmers, access to information, household income, and farming experience. Major constraints faced by tribal farmers included poor access to modern practices, low harvest prices, and insufficient credit supply. Livelihood diversification emerged as crucial for sustainable livelihoods, improving household income and resilience. Modern agricultural practices have boosted crop productivity, supporting food security. Thus, promoting livelihood diversification among tribal farmers is essential for improving their resilience and income sustainability. This can be achieved by offering training, market access, and financial support for activities like poultry and livestock rearing, as well as non-farm enterprises such as homestead gardening. Policy recommendations include encouraging rice production due to its profitability, promoting off-farm economic activities, ensuring access to quality inputs at reasonable prices, and facilitating access to credit with lower interest rates. Better communication infrastructure

in geographically diverse areas like the CHT is essential for marketing facilities. Measures to reduce marketing margins and enhance market information dissemination are also proposed. Extension services should assist farmers in adopting agricultural technologies, while encouragement of off-farm activities can provide a more robust livelihood structure.

Funding: This research received no external funding

Conflicts of Interest: The authors declare no conflicts of interest.

Ethics Statement: The study was approved by the Sylhet Agricultural University Research System (SAURES) in accordance with the rules and regulations of the Sylhet Agricultural University, Sylhet, Bangladesh.

Compliance with Ethical Standards:

- This original research manuscript has not been published and is not under consideration for publication elsewhere.
- All the compliances and ethical Standard was followed during the research stages, such as data collection and analysis.
- The authors declare that they have no conflicts of interest.
- All procedures performed in studies involving human participants were in accordance with the ethical standards of the institutional and/or national research committee.
- Informed consent was obtained from all individual participants involved in the study.

Data availability: The data presented in this manuscript are available on request from the corresponding author.

Code availability: Not Applicable

References

Afrad, M. S. I. (2002). *Farmers' Attitude towards Vegetable Cultivation in Dumki Upazila of Patuakhali District*. Bangladesh Agricultural University, Mymensingh.

Ahmed, H., & Ahmed, M. (2023). Influencing Factors on Adoption of Modern Agricultural Technology in Developing Economy Countries. *Developing Country Studies*, *March*. https://doi.org/10.7176/dcs/13-2-01.

Ahmed, T., Bhandari, H., Gordoncillo, P. U., Quicoy, C. B., & Carnaje, G. P. (2015). Diversification of rural livelihoods in Bangladesh. *Journal of Agricultural Economics and Rural Development*, *2*(2), 32–38.

Akudugu, M. A., Nkegbe, P. K., Wongnaa, C. A., & Millar, K. K. (2023). Technology adoption behaviors of farmers during crises: What are the key factors to consider? *Journal of Agriculture and Food Research*, *14*(January), 100694. https://doi.org/10.1016/j.jafr.2023.100694.

Alam, J. (2004). Analysis .of crop sector diversification in Bangladesh. *Journal of Bangladesh Agricultural University*, *2*(2), 375–386.

Barau, A. A., Afrad, M. S. I., Rahman, M. S., & Rahman, M. A. (2019). Indigenous peoples'

livelihood practices in south-eastern Bangladesh and the question of sustainability. *Sustainable Development*, *27*(4), 809–816. https://doi.org/10.1002/sd.1924.

Barkat, A., Suhrawardi, G. M., Osman, A., Sobhan, M. A., & Rafique, R. B. (2017). *Agricultural Production Practices in Chittagong Hill Tracts* (Issue August 2021). Human Development Research Centre.

BBS. (2021). *Statistical Yearbook Bangladesh 2021* (Issue June). Bangladesh Bureau of Statistical Division. Ministry of Planning. Government of the People's Republic of Bangladesh. www.bbs.gov.bd

Bokhtiar, S. M., & Samsuzzaman, S. (2023). *A Development Trajectory of Bangladesh Agriculture: From Food Deficit to Surplus*. Bangladesh Agricultural Research Council, Dhaka, Bangladesh. www.barc.gov.bd

Chakma, N., & Maitrot, M. (2017). How ethnic minorities became poor and stay poor in Bangladesh: A qualitative enquiry. In *Fcdo* (Issue December). https://doi.org/10.13140/RG.2.2.11954.25289.

Ellis, F. (1998). Household strategies and rural livelihood diversification. *Journal of Development Studies*, *35*(1), 1–38. https://doi.org/10.1080/00220389808422553.

FAO. (2017). The future of food and agriculture: Trends and challenges. In *Food and Agriculture Organization of the United Nations Rome*. https://www.fao.org/3/i6583e/i6583e.pdf.

Gallardo, R. K., & Sauer, J. (2018). Adoption of Labor-Saving Technologies in Agriculture. *Annual Review of Resource Economics*, *10*, 185–206. https://doi.org/10.1146/annurev-resource-100517-023018.

Gautam, Y., & Andersen, P. (2016). Rural livelihood diversification and household well-being: Insights from Humla, Nepal. *Journal of Rural Studies*, *44*, 239–249. https://doi.org/10.1016/j.jrurstud.2016.02.001.

Ghosh, M. K., Md. Rezaul Karim, Orpita, F. U. R., Mst. Lamia Jahan, Simi, F., & Sony, M. A. A. (2023). Exploring Farmers Perspectives on Modern Agricultural Technology: A Study in Chapainawabganj District. *European Journal of Agriculture and Food Sciences*, *5*(4), 19–27. https://doi.org/10.24018/ejfood.2023.5.4.689.

Hill, L., & Kau, P. (1973). Application of Multivariate Probit to a Threshold Model of Grain Dryer Purchasing Decisions. *American Journal of Agricultural Economics*, *55*(1), 19–27.

Hossain, M. A. (2011). An overview on shifting cultivation with reference to Bangladesh. *Scientific Research and Essays*, *6*(31), 6509–6514. https://doi.org/10.5897/SRE11.1282.

Islam, M. A., Hossain, M. N., Chokraborti, S. S., Rahman, S., Tasnim, A., & Al Zabir, A. (2018). Socio-economic Profile of Goat Rearing Farmers and Their Management Practices in Sylhet, Bangladesh. *Journal of Agriculture and Ecology Research International*, *15*(4), 1–10. https://doi.org/10.9734/jaeri/2018/43818.

Jannat, A., Islam, M. M., Alamgir, M. S., Al Rafi, D. A., & Ahmed, J. U. (2019). Impact assessment of agricultural modernization on sustainable livelihood among tribal and non-tribal farmers in Bangladesh. *GeoJournal*, *86*(1), 399–415. https://doi.org/10.1007/s10708-019-10076-4.

Kabir, M. H., & Rainis, R. (2015). Adoption and intensity of integrated pest management (IPM) vegetable farming in Bangladesh: an approach to sustainable agricultural development. *Environment, Development and Sustainability*, *17*(6), 1413–1429. https://doi.org/10.1007/s10668-014-9613-y.

Mariyono, J. (2018). Profitability and Determinants of Smallholder Commercial Vegetable

Production. *International Journal of Vegetable Science*, *24*(3), 274–288. https://doi.org/10.1080/19315260.2017.1413698.

Martin, S. M., & Lorenzen, K. (2016). Livelihood Diversification in Rural Laos. *World Development*, *83*, 231–243. https://doi.org/10.1016/j.worlddev.2016.01.018.

Miah, M. D., Chakma, S., Koike, M., & Muhammed, N. (2012). Contribution of forests to the livelihood of the Chakma community in the Chittagong Hill Tracts of Bangladesh. *Journal of Forest Research*, *17*(6), 449–457. https://doi.org/10.1007/s10310-011-0317-y.

Nahar, A., Ali Akbar, M., Biswas, J. C., Gafur, A., Farid Uddin, M., Rashid, S., Abdul Mannan Mollah, M., Sanue Marma, M., Mong Marma, T., Swiy Shing Marma, A., Kamrul Islam, M., Neogi, M., & Hamid, A. (2020). Household Demography and Food Security of Jhum Farmers in Bandarban District, Bangladesh. *Journal of Applied Agricultural Economics and Policy Analysis*, *3*(1), 8–14. https://doi.org/10.12691/jaaepa-3-1-2.

Nath, T. K., Inoue, M., & Chakma, S. (2005). Shifting cultivation (jhum) in the Chittagong Hill Tracts, Bangladesh: examining its sustainability, rural livelihood and policy implications. *International Journal of Agricultural Sustainability*, *3*(2), 130–142. https://doi.org/10.1080/14735903.2005.9684751.

Patr, M., Begum, S., & Deka, B. C. (2014). Problems and prospects of traditional pig farming fsor tribal livelihood in Nagaland. *Indian Research Journal of Extension and Education*, *14*(4), 6–11. http://seea.org.in/ojs/index.php/irjee/article/download/236/235.

Punitha, P., Chauhan, J., Singh, R., & Singh, R. J. (2018). Livelihood diversiication of of Manipur: a constraint analysis. *Journal Article: Indian Research Journal of Extension Education*, *18*(1), 22–27.

Rasul, G. (2015). A strategic framework for sustainable development in the Chittagong Hill Tracts of Bangladesh. In *ICIMOD Working Paper* (Issue 2015/3). https://doi.org/10.13140/RG.2.1.1196.2963.

Rasul, G., & Gurung, P. (2024). Unlocking the potentials of sustainable livelihoods in Chattogram Hill Tracts of Bangladesh. *Nature-Based Solutions*, *5*(July 2023), 100108. https://doi.org/10.1016/j.nbsj.2023.100108.

Rozario, S. R., Mirza, A. B., & Akhter, F. (2022). *The impacts of human-induced climate change are exacerbating social and economic inequalities of indigenous peoples – A case study from Bangladesh*. International Centre for Climate Change and Development. https://www.preventionweb.net/news/impacts-human-induced-climate-change-are-exacerbating-social-and-economic-inequalities.

Saha, B., & Bahal, R. (2010). Livelihood diversification pursued by farmers in West Bengal. *Indian Research Journal of Extension Education*, *10*(2), 1–9.

Sarker, M. R., Galdos, M. V., Challinor, A. J., & Hossain, A. (2021). A farming system typology for the adoption of new technology in Bangladesh. *Food and Energy Security*, *10*(3), 1–18. https://doi.org/10.1002/fes3.287.

Sen, B., & Ali, Z. (2015). Ending extreme poverty in Bangladesh during the Seventh Five Year Plan: trends, drivers and policies. In *Background paper for the preparation of the Seventh Five Year Plan. General Economics Division, Planning Commission*. https://doi.org/http://dx.doi.org/10.3362/9781780449463.002.

Start, D. (2001). The Rise and Fall of the Rural Non-farm Economy: Poverty Impacts and Policy Options. *Development Policy Review*, *19*(4), 491–505. http://dx.doi.org/10.1111/1467-

7679.00147.

Tailor, R., Meena, G. L., Sharma, L., & Sharma, F. L. (2012). Constraints faced by the tribal farmers in dairy farming in Udaipur district. *Rajasthan Society of Extension Service*, *20*, 187–189.

UNICEF. (2019). *Many Tracts One Community: UNICEF'S Work in the Chittagong Hill Tracts*. www.unicef.org.bd.

Wongnaa, C. A., Awunyo-Vitor, D., & Andivi Bakang, J. E. (2018). Factors affecting adoption of Maize production technologies: A study in Ghana. In *Journal of Agricultural Sciences - Sri Lanka* (Vol. 13, Issue 1, pp. 81–99). https://doi.org/10.4038/jas.v13i1.8303

Wongnaa, C. A., & Babu, S. (2020). Building resilience to shocks of climate change in Ghana's cocoa production and its effect on productivity and incomes. *Technology in Society*, *62*(June), 101288. https://doi.org/10.1016/j.techsoc.2020.101288.

World Bank. (2017). *Bangladesh continues to reduce poverty but at a slower pace*. https://www.worldbank.org/en/news/feature/2017/10/24/bangladesh-continues-to-reduce-poverty-but-at-slower-pace#:~:text=Since 2010%2C 8 million Bangladeshis,pace of poverty reduction slowed.

Wu, F. (2022). Adoption and income effects of new agricultural technology on family farms in China. *PLoS ONE*, *17*(4 April), 1–19. https://doi.org/10.1371/journal.pone.0267101.

Xaba, B. G., & Masuku, M. B. (2013). Factors Affecting the Productivity and Profitability of Vegetables Production in Swaziland. *Journal of Agricultural Studies*, *1*(2), 37. https://doi.org/10.5296/jas.v1i2.3748.
