# Supplementary material for: The role of livelihood diversification in agricultural modernization among tribal farmers in Bangladesh: A binary logistic model approach
Source: PLoS One. 2025 Feb 25;20(2):e0318457. doi: 10.1371/journal.pone.0318457 (PMC11856501; doi:10.1371/journal.pone.0318457)
Supplement: Attachment — Submitted filename: Reviewer_response_R1_20241124.docx [file pone.0318457.s003.docx]

**The Role of Livelihood Diversification in Agricultural Modernization Among Tribal Farmers in Bangladesh: A Binary Logistic Model Approach**

Manuscript ID: PONE-D-24-36026

**Responses to Reviewer’s Comments**

Thank you so much for considering the paper based on the proposed revisions. The authors gave full consideration of making the corrections advised after thoroughly reading the comments. The point-to-point questions and answers are given below. Besides, please note that the changed areas are based on the reviewer's comments.

**Reviewer-1: Comments**

The study explored livelihood diversification and its impact on the adoption of modern agricultural techniques. The results indicated that the decision to adopt modern agricultural practices was significantly correlated with livelihood diversification, the age of the farmers, access to information, household income, and farming experience. Major constraints faced by tribal farmers included poor access to modern practices, low harvest prices, and insufficient credit supply. Livelihood diversification emerged as crucial for sustainable livelihoods, improving household income and resilience. Modern agricultural practices have boosted crop productivity, supporting food security. Thus, promoting livelihood diversification among tribal farmers is essential for improving their resilience and income sustainability. This can be achieved by offering training, market access, and financial support for activities like poultry and livestock rearing, as well as non-farm enterprises such as homestead gardening. Policy recommendations include encouraging rice production due to its profitability, promoting off-farm economic activities, ensuring access to quality inputs at reasonable prices, and facilitating access to credit with lower interest rates.

Overall article is written well, however, the English language needs to be improved.

**Response:** Thank you for your appreciation of our manuscript. We acknowledge your comment regarding the need to improve the English language. Accordingly, we have thoroughly reviewed the manuscript to enhance the grammar, sentence structure, and overall readability.

**Reviewer-2: Comments**

The paper can be accepted after the revision. The detailed comments are provided inside the documents.

The authors are suggested to give line numbers while revising the paper. It is difficult to provide the comments.

**Response:** Thank you for your valuable feedback on our manuscript. We greatly appreciate the time and effort you have invested in reviewing our work. Regarding your suggestion to include line numbers in the revised version of the manuscript, we have ensured that line numbers are now added throughout the document to facilitate easier reference and comment. We apologize for any inconvenience caused by their absence in the original submission.

The author needs to include the data availability consent for crosschecking the data used in the manuscript.

**Response:** In response to your suggestion regarding data availability consent, we have now included a dedicated Data Availability Statement section in the manuscript. This section provides details about how and where the data used in our study can be accessed for verification and further research.

The research content of the manuscript seems to deviate from the goal in a few places, especially in the discussion part. Please revise accordingly.

**Response:** Thank you for your constructive feedback on our manuscript, particularly regarding the alignment of the research content with the study’s goals. We appreciate your valuable observation and have carefully revised the manuscript to address this concern. In the revised manuscript, we have thoroughly reviewed and refined the discussion section to ensure it remains focused on the study’s objectives. We have streamlined the arguments to directly address the core research questions, emphasizing the relationship between livelihood diversification, modern agricultural practices, and their impact on sustainability and resilience. We revised the discussion to clearly connect key findings, such as the role of livelihood diversification in sustainable agriculture, with actionable implications and policy recommendations.

**Additional Comments from the Manuscript:**

Page 13: Loan for what?

**Response:** Thank you for your insightful observation regarding the source and purpose of loans mentioned in the manuscript.

In response to your query about the specific purposes for which loans were taken, we have clarified this in the revised manuscript. In the study area, most people utilized credit from the Bangladesh government’s social welfare program, Ektee Bari Ektee Khamar (One House One Farm), and a smaller proportion took out loans from banks. These loans were primarily used for agricultural activities (Purchasing inputs such as seeds, fertilizers, and pesticides, renting machinery) and non-Non-Agricultural Income-Generating Activities (establishing small businesses such as shops or handicrafts, expanding non-farm enterprises like homestead gardening or livestock rearing).

Page 14: may be farm family with more diversified option earn more income

**Response:** Thank you for your valuable observation regarding the relationship between income and livelihood diversification.

In our study, we defined livelihood diversification as the extent to which households engage in multiple income-generating activities beyond their primary occupation. Households with more sources of income, such as combining crop farming with livestock rearing, non-farm businesses, or wage labor, are inherently more diversified due to their engagement in varied economic activities. This approach aligns with the established definitions of diversification in the livelihood literature, where diversification is measured by the variety and number of income streams. We have revised the manuscript to clarify this reasoning and have also elaborated on the potential causal pathways connecting diversification, income stability, and overall household earnings.

Page 15: Can the authors convers TK to USD as this is an International Journal or somewhere mention 1Tk is how much USD

**Response:** Thank you for your suggestion regarding the conversion of Bangladeshi Taka (Tk) to USD. To improve the manuscript’s clarity for an international audience, we have taken the following steps:

Wherever monetary values are mentioned, we have provided their equivalent in USD using the prevailing exchange rate during the study period. For instance, the conversion rate (e.g., "1 USD = BDT 83.5") has been included in the revised version of the manuscript to serve as a reference.

Page 16: Rephrase this sentence "The perching method hardly costs anything"

**Response:** Thank you for your feedback. In response to your comment, we have revised the sentence to better convey the intended meaning. The revised sentence now reads: "The perching method incurs minimal to no cost." This revision clarifies that the method is virtually cost-free or requires negligible expenditure.

Page 17: Please consider revising the unit to USD

**Response:** Thank you for your suggestion regarding the conversion of Bangladeshi Taka (Tk) to USD. To improve the manuscript’s clarity for an international audience, we have taken the following steps:

Wherever monetary values are mentioned, we have provided their equivalent in USD using the prevailing exchange rate during the study period. For instance, the conversion rate (e.g., "1 USD = BDT 83.5") has been included in the revised version of the manuscript to serve as a reference.
